# Review of Cisplatin and Oxaliplatin in Current Immunogenic and Monoclonal Antibodies Perspective

**DOI:** 10.14740/wjon830w

**Published:** 2014-06-25

**Authors:** Rao Khalid Mehmood, Jody Parker, Shakil Ahmed, Eyas Qasem, Ahmed A Mohammed, Muhammed Zeeshan, Ernest Jehangir

**Affiliations:** a Betsi Cadwaladr University Health Board, Department of Surgery, Ysbyty Glan Clwyd, Rhyl, North Wales, LL18 5UJ, UK; b The Royal Liverpool and Broadgreen University Hospitals NHS Trust, Prescot Street, Liverpool, L7 8XP, UK; c Acute University Hospitals NHS Trust, Cumberland Infirmary Carlisle, Newtown Rd, Carlisle, Cumbria, CA2 7HY, UK

**Keywords:** Cisplatin, Oxaliplatin, Monoclonal antibodies, Combination therapy

## Abstract

Platinum-based chemotherapy made a paradigm shift in the treatment of different cancers initially; however, the success of these agents may have reached the peak as researchers have tried different combination regimes in different trials without having major differences in the end results. New frontiers of research were opened up firstly with this discovery that conventional chemo-radiation therapy can induce immunological cell death by recruiting high-mobility group box 1 (HMGB1) protein which triggers the T cell immunity and secondly monoclonal antibodies agents which were regrettably not effective as “monotherapy”; however, the combination with conventional chemotherapy had demonstrated good results. Different monoclonal antibodies and conventional chemotherapeutic combination regimes are currently in use and researchers are trying different other combinations as well to glean the maximum benefits from them. Several strategies conferring resistance to platinum compounds have been identified, but there is still significant research required to achieve full understanding of these resistance mechanisms to overcome the ineffectiveness or toxicities of platinum compounds. It seems reasonable in the current perspective when conventional chemotherapeutic agents exhibited immunogenic cell death and they are currently in use with monoclonal antibodies to revisit the platinum agent’s pharmacology. This may discover new basis for combination chemotherapy with monoclonal antibodies which may improve the current cancer treatments by opening new vistas for newer combination regimes with less toxicity and better efficacy. In this article we review the pharmacologies of both cisplatin and oxaliplatin in the drug development perspectives and explore the possible association of these drugs with monoclonal antibodies.

## Introduction

Cisplatin is a standard treatment in many cancers for example, advanced germ cell tumors which were previously considered almost fatal [1-3]. However side effects and acquired resistance limited its usage [4] and it led to the efforts to develop newer compounds which should be highly effective and less susceptible to develop resistance. Therefore a new platinum compound oxaliplatin, which is characterized by its 1, 2-diaminocyclohexane (DACH) carrier ligand was developed. It lacked cisplatin’s nephrotoxicity and is active in some cisplatin resistant tumors [5, 6].

Oxaliplatin has got its own side effects, however, like peripheral neuropathy and other toxicities. Different combinations of oxaliplatin with other chemotherapeutic agents are currently in use for example in colorectal cancers. However researcher could not bring an “ideal” combination with lesser toxicity and broader cytotoxicity. Conventionally efforts to find the solutions to reduce platinum drug resistance and discover newer agents were considered the way forward. However the advent of monoclonal antibodies drugs (MADs) and their combination with conventional platinum and other chemotherapeutic agents had opened new frontiers to the researchers.

MADs such as cetuximab, trastuzumab and bevacizumab are used to treat colorectal, breast and lung cancers and others [7, 8]. They inhibit key proteins associated with tumor development. Bevacizumab for example, targets and blocks a protein called vascular endothelial growth factor (VEGF). This protein helps cancer cells in angiogenesis, i.e. to develop new blood vessels. When this protein is blocked by bevacizumab, tumor vascular endothelial cell proliferation stops, and oxygen and nutrient supplies are hampered which shrinks it and inhibits its growth [7, 9]. These agents, however, carry their own toxicities and resistance [10, 11].

The concept that conventional chemoradiotherapy can cause immunological cell death by recruiting high-mobility group box 1 (HMGB1) protein which triggers the T-cell-induced immunity, has potentially raised hopes that these findings can be translated in clinical practice which may improve the cancer management. Therefore the designing of combination chemotherapy with monoclonal antibodies may become effective in overcoming the problems of platinum resistance and toxicities and may increase their cytotoxicity profile.

In this article we aim to discuss the pharmacology of cisplatin and oxaliplatin. This “revisiting” may help in developing a better understanding of these time-tested drugs in perspectives of their immunogenic cell death and monoclonal antibodies which may help in devising new future trials.

## Cisplatin and Its Effects on DNA

**Figure 1:**
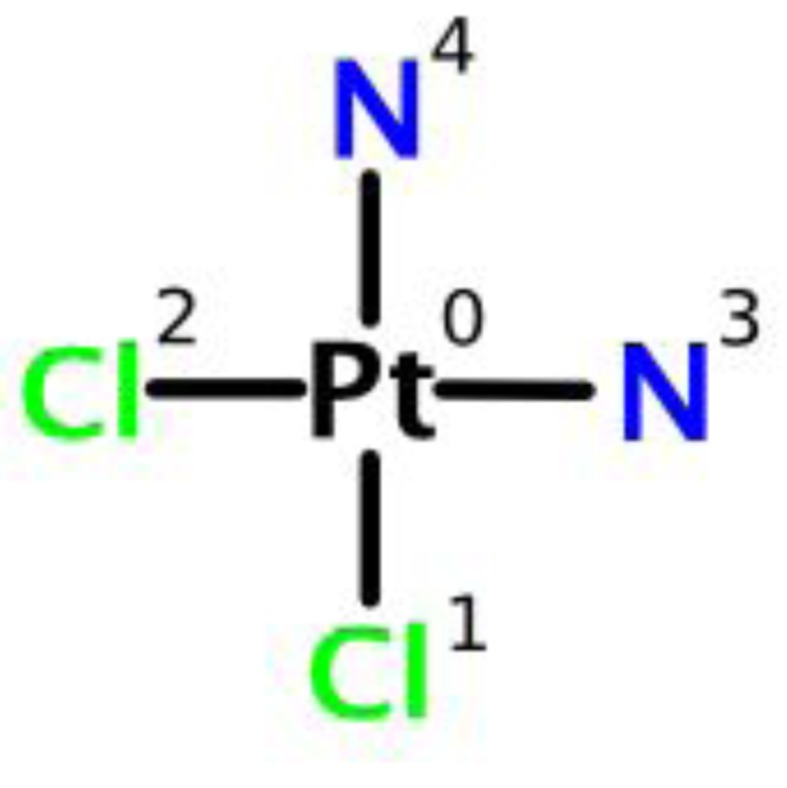
Chemical structure of cisplatin.

Cisplatin is a heavy metal complex containing a central atom of platinum surrounded by two chloride atoms and two ammonia molecules in the cis position. It is soluble in water or saline [12] (Fig. 1).

Chloride atoms of cisplatin are displaced in a chemical reaction by nucleophiles, such as water or sulfhydryl groups, rather than enzyme-catalyzed metabolism. Cisplatin does not immediately and reversibly bind with plasma proteins, which are characteristics of normal drug-protein binding for other drugs. The platinum component of cisplatin binds to several plasma proteins, including albumin, transferrin or gamma globulin over period of time and irreversibly [12]. Three hours after a bolus injection 90% of the plasma platinum is still protein bound. The complexes between albumin and the platinum from cisplatin do not dissociate to a significant extent and are slowly eliminated with a minimum half-life of 5 days or more [12].

### Effects on DNA

It is generally agreed that cisplatin exhibits its cytotoxic effects mainly through genomic DNA (gDNA) binding in the cell nucleus. The main effect happens after binding with DNA which makes DNA replication or transcription processes futile and causes cancer cell death [13, 14].

Inside a cell, cisplatin undergoes hydrolysis, producing the highly reactive charged platinum complex (Pt(NH_3_)_2_ClH_2_O)^+^. This complex after further hydrolysis eventually binds to DNA bases through the N7 atom preferably with guanine. The overall effect of this DNA crosslinkage mechanism causes interference with cell division/replication by mitosis. Although the damaged DNA initiates repair mechanism, it instead of repairing activates apoptosis when repair process proves futile [14, 15].

Cisplatin forms different adducts with DNA which are structurally different from each other. At the start “monofuctional” DNA adducts are formed which further react to produce “intra-strand” or “inter-strand” DNA adducts [16]. It has been discovered that 1, 2-d (GpG) intra-strand makes around 60-65% and 1, 2-d (ApG) intra-strand makes around 20-25% of cisplatin DNA adducts. The 1, 3-intra-strand forms a small percentage of cisplatin DNA adducts [17]. It has been reported that cisplatin forms adducts with mitochondrial DNA and it also induces DNA protein crosslinks [18].

All three types of cisplatin DNA adducts are involved in unwinding of DNA helix at different degrees, for example, 1, 2-d (GpG) and 1, 2-d (ApG) intra-strand unwind DNA by 13°, while the 1, 3-d (GpXpG) intra-strand unwind DNA by 23° respectively. However, in spite of differences in unwinding degrees of DNA helix their bending capacity of DNA helix remains the same (32 - 35°) [19]. These unwinding and bending processes of cancer cell DNA make them irreparable which results in cancer cell death. Which cisplatin DNA adducts or cisplatin combination with cellular proteins or other mechanisms play a major role in cancer cell death, still needs more research to settle the debate. However currently available evidence supports the idea that 1, 2-intra-strand DNA adducts play a major role in its cytotoxicity because transplatin is unable to form these kinds of adducts [19] and more importantly these adducts are difficult to be removed from DNA by nucleotide excision repair (NER) than 1, 3-intra-strand adducts [20, 21]. Furthermore 1, 2-d (GpG) or 1, 2-d (ApG) adducts demonstrate the highest affinity for HMGB1. It is therefore postulated that certain specific type HMG proteins may take part in the cellular processing of these 1, 2-intra-strand formed by cisplatin [22] which could have made these adducts more cytotoxic rather than other types; however, the importance of the other minor adducts and DNA protein adducts should not be overlooked in the overall cytotoxicity profile of cisplatin [22].

Comparatively oxaliplatin adducts bind HMGB1 much less avidly than that of cisplatin adducts [23].

It has been demonstrated that cisplatin forms a high amount of adducts in mitochondrial DNA (mtDNA) which is rather believed naked because it lacks histones [14, 24, 25]. Moreover, mitochondria are unable to carry out NER, a major pathway for removing cisplatin-DNA adducts [26]. Therefore this pathway may be a major contributor in cellular death and an important contributor in cisplatin’s toxicity. Before binding of cisplatin occurs to genomic or mitochondrial DNA a loss of chloride group is needed. Since the higher chloride concentration in extracellular fluids impedes the formation of mono- and diaquo cis-Pt (II) species in which one or both chloride groups are replaced by water molecules [14]. In contrast, within the cell, the chloride concentration is low, the hydrolysis of cisplatin adducts happens quite effectively and both of its chloride leaving groups are replaced by water molecules, which results in the formation of aquo species and (Pt(H_2_O)_2_(NH3)_2_)^2+^ cation is formed. This molecule carries two water molecules, diaquo species, which make it more reactive towards nucleophilic centers of biomolecules, and cisplatin’s cytotoxicity may arise from these diaquo species reactions with DNA [14, 27].

## Oxaliplatin and Its Effects on DNA

Because of the side effects of cisplatin especially its renal and GI side effects attempts were made to introduce new platinum drugs which carry less side effects and be more cytotoxic than cisplatin. It resulted in the development of carboplatin which replaced it in many chemotherapeutic regimens. However, efforts were continued, and nedaplatin and oxaliplatin were introduced. Oxapliatin showed no cross-resistance with cisplatin and carboplatin and did not exhibit similar nephrotoxicity (< 1%). Its ototoxicity is reported < 3%. However oxapliatin carries its own side effects, sensory and motor neuropathy [28].

**Figure 2:**
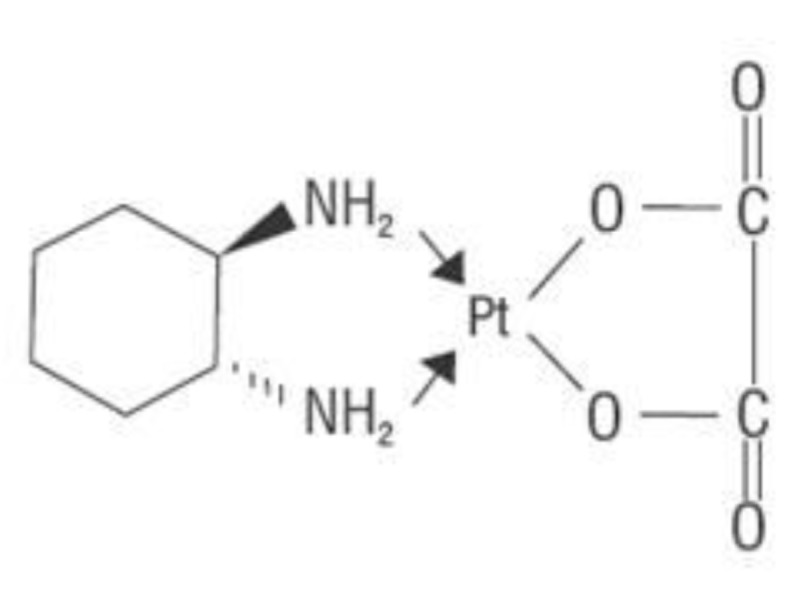
Chemical structure of oxapliatin.

Oxaliplatin is an organoplatinum complex in which the platinum atom is complexed with 1, 2-DACH (Fig. 2), and with an oxalate ligand as a “leaving group” (Fig. 2). A “leaving group” or labile atom is an atom or a group of atoms that is displaced from the stable component taking with it the bonding electrons. Oxaliplatin undergoes nonenzymatic conversion in physiologic solutions to active derivatives via displacement of the labile oxalate ligand. Several transient reactive species are formed, including monoaquo and diaquo DACH platinum, which covalently bind with macromolecules. Initially, only mono-adducts are formed but eventually oxaliplatin attaches simultaneously to two different nucleotide bases, resulting in DNA crosslinks [28]. Both inter- and intra-strand Pt-DNA adducts or crosslinks are formed [29]. These crosslinks are formed between the N7 positions of two adjacent guanines (GG), adjacent adenine-guanines (AG), and guanines separated by an intervening nucleotide (GNG). These crosslinks inhibit DNA replication and transcription. Oxaliplatin cytotoxicity is cell-cycle nonspecific [30].

The precise mechanism of action of oxaliplatin is unclear and is largely extrapolated from cisplatin and other DACH compounds [5]. Both cisplatin and oxaliplatin are DNA alkylating agents forming platinated intra-strand and inter-strand crosslinks [31]. Intra-strand crosslinks contribute significantly to cisplatin’s cytotoxicity but seems less important in oxaliplatin [32]. The DACH side chain of oxaliplatin is thought to enhance cytotoxicity and abolish cross-resistance between oxaliplatin and other platinum compounds.

Generally the cytotoxicity of platinum drugs is related to the saturation of the cellular ability to repair platinum DNA adducts or the abilities of these DNA adducts to stop new cellular DNA synthesis or repair. However less number of oxapliatin adducts is found more effective. Therefore mechanisms other than DNA adducts are involved in cellular death [28].

The synergism has been demonstrated between oxaliplatin and 5-fluorouracil. Anti-proferative properties of oxaliplatin and 5-fluorouracil increased *in vitro* and *in vivo* in combination greater than either compound alone in several tumor models like colon, breast and leukemia [30].

There is evidence suggesting that DNA adducts are not the sole mechanism of platinum drug cytotoxicity. Oxaliplatin, for example, acts in leukemia cells cultures at different levels, and it interferes with RNA and cellular proteins. It also forms bondage with sulfhydryl groups in cellular proteins which make them inactive and impair with cellular functions [33].

Oxaliplatin’s DACH ligand is more water soluble and bulkier than amino group of cisplatin or carboplatin that results in greater deformation of cancer cell DNA by steric hindrance by adduct formation which may explain oxaliplatin’s greater cytotoxicity in comparison with cisplatin [9].

Moreover, because of the DACH ligand, mismatch repair (MMR) complex is unable to bind oxaliplatin DNA adducts secondary to its pronounced steric distortion of the DNA structure [3] which may further increases its cytotoxicity. DNA repair enzymes are covalently bound with oxaliplatin which impairs their functions [33]. If DNA damage is substantial and could not be repaired, it may ultimately lead to the activation of apoptotic pathways and cellular death [32].

## Cisplatin and Oxaliplatin Mechanisms of Action in General

### NER system

Lesions in the DNA whether inflicted by endogenous or exogenous sources are repaired by NER which is extremely sophisticated and versatile in its actions and approach in removing these damaging agents and restoring the normal state of DNA [34]. NER is further subdivided into two types global genomic NER (GG-NER) and transcription-coupled NER (TC-NER), depending on their mode/ability in identifying the damaged site. Cisplatin DNA lesions are mainly repaired by TC-NER pathway. However, no significant difference between the repair of 1, 2-d (G*pG*)-Pt adducts type of cisplatin and oxaliplatin was observed [35].

### Transcription-coupled repair (TCR)

TCR is a sub pathway of NER. The efficiency of DNA repair varies partly because it is attached to transcription. DNA damaged sites are identified by stalled or paused RNA polymerases which recruit repair proteins in a process called transcription-coupled nucleotide excision repair. It has been demonstrated that stalled or paused transcription complexes start a damage detection process which results in strand specific lesion repair [36].

TCR identifies the damaged site on DNA by stalled polymerases and these lesions are removed preferentially [37]. It has been demonstrated that cells deficient in TCR are more sensitive to cisplatin in comparison to the cells which are not deficient in TCR [38]. TCR mechanism of repair has not fully understood so far and needs further investigation especially in establishing its role in processing Pt-DNA damage.

### Pt-DNA adducts inhibit RNA elongation

Studies have demonstrated that Pt-DNA adducts stop process of transcription *in vitro* in cell-based assays [35, 39]. These results have been confirmed in recently reproduced experiments in live cells by using luciferase assays [35]. Currently one of the hypothesis suggested that inhibition of transcription process by the DNA adducts in the living cells may happen because of the blockage of RNA elongation [36].

### Repair of Pt-DNA adducts by other mechanisms

Studies have recently identified that cells can bypass the transcription processes even in the presence of viable and working NER system to repair the platinum DNA adducts and also in the NER deficient XPF cells, and the process of transcription may recover although it takes time to do so to remove platinum blockage. For example, mismatch repair removes the platinum block during the long time course of the luciferase assay [35, 40]. These observations suggest that there are other mechanisms present which may not be modeled by the currently available conventional *in vitro* assays but which needs further investigation.

### Protein binding with DNA adducts

Cisplatin DNA adducts bind tightly and selectively with HMGB1 which influences its mechanism of action [41].

## Other Mechanisms for Cytotoxicities of Cisplatin and Oxaliplatin

DNA damage to the cell can happen with several mechanisms which may cause cell death or these damages can be repaired by the cell to survive. One of the suggested pathways of apoptosis is that platinum DAN adducts block the process of transcription by stalling RNA polymerases which results in apoptosis through p53-dependent and p53-independent pathway [42].

### Envisaging tailored platinum chemotherapy based on Pt-DNA adducts processing

Studies have identified that how certain specific type of DNA platinum blocks transcription process in live cells. The extent of this blockage depends on the type of the lesion and the ability of the DNA adducts to block the passage of polymerase II; however, all these steps can be reversed by NER system to restore transcription.

Other mechanisms of DNA repair have been mentioned earlier on other than NER. By processing the platinum DNA adducts in the real cellular environment mechanism of action of major platinum drugs can be elucidated and may offer the potential benefit to select the platinum drug for the treatment of cancer which is based on its capability to stop transcription process “from a globally or site-specifically modified probe in live cells derived from the cancer tissue” [35].

### Excision repair cross complementing 1 (ERCC1) and xeroderma pigmentosum A (XPA)

In cisplatin resistant cells NER activity is increased which appears to be dependent on expression of ERCC1 and XPA. A mutation on XPA can prevent NER interaction which abolished DNA repair response [43]. For example, testicular germ cell tumors with low XPA can restore their cisplatin adduct removing ability after its increased expression. These cells have also demonstrated increased residual oxaliplatin DNA adducts with greater cytotoxic effect [44]. ERCC1 is over-expressed in cisplatin resistant cells both *in vitro*. Arnould et al found that increased ERCC1 expression correlated to a lower residual level of cisplatin DNA adducts and reduced cytotoxicity [45]. Although ERCC1 levels are predictive of oxaliplatin cytotoxicity in many cell lines, these levels do not correlate with oxaliplatin DNA adducts [46, 47].

#### Post replication repair (PRR)

PRR is the repair of damage to the DNA that takes place after replication. As the presence of gaps or discontinuities in the DNA can be lethal, DNA repair after replication is a major mechanism of DNA damage tolerance [14, 48]. DNA enzymes which are involved in the PRR are able to work for DNA synthesis on its leading strand in the presence of platinum adducts, which demonstrate that presence of these platinum adducts may not act as an absolute hinderer to DNA replication. However the presence of DNA platinum adducts may affect replicative enzyme performance and accuracy. In these circumstances PRR is vital for the survival of the cells, otherwise the gaps and discontinuities will cause cell death.

Although PRR normally takes place primarily during cell replication, in cisplatin resistant cell lines, PRR was found active during non-replication phase as well which may signify that PRR may be involved in cisplatin’s resistance. Many replicative enzymes are involved in PRR including BRCA2, BRCA1 and different polymerases. It is still not clear which type of polymerases is involved in PRR. However, recently in an HCT-8 human colon tumor cell line, high levels of polymerase β were found which is in line with cellular resistance to oxaliplatin [28, 49].

#### MMR

The genetic accuracy of DNA polymerases is high but there is still a small percentage of mismatched base pairs in newly synthesized DNA which may result in mutation if not corrected by MMR. Therefore MMR is DNA mismatch repair pathway which corrects base mispairs and small strands. MMR consists of six different proteins hMLH1, hMLH2, hPMS2, hMSH2, hMSH3 and hMSH6 genes. Resistance to cisplatin has been reported to defects in one of these proteins most probably in hMLH1 in combination with others [28, 50]. MLH1 works as a damage recognition unit like high-mobility group protein (HMG1), which is in line with its observed role in cell cycle regulation and incitation of apoptosis [28, 51].

*In vitro* studies demonstrated that MMR appears insignificant in oxaliplatin-induced DNA-damage repair process. However it works as an essential mechanism in cisplatin and carboplatin adduct repair. This may be because of different configurational distortion of the oxaliplatin DNA adducts, and the presence of DACH ligand in it may have proved important in MMR’s failure to detect these adducts in comparison to cisplatin adducts [28].

#### Damage recognition proteins

Replicative bypass repairs damaged DNA and its specificity is determined by DNA polymerases, MMR and damage recognition proteins (DRPs) [52]. Only 5-15% of sporadic tumors are MMR defective [53] suggesting other mechanisms influence specificity of replicative bypass. DRPs bind to platinum DNA adducts decreasing replicative bypass either by removing new DNA opposite to these adducts with MMR or by blocking trans-lesion synthesis beyond the DNA adducts [54]. More than 20 DRPs exist which bind with different affinities to cisplatin and oxaliplatin adducts [44, 55, 56].

DRPs influence sensitivity to DNA adducts which blocks NER [55], sequestering TF’s or activating signal transduction pathways leading to cell cycle arrest or apoptosis [57]. Characterization of DNA repair specificity is important in providing testable models for understanding how DNA repair pathways influence platinum drugs resistance [44].

#### Apoptosis

The Bcl-2 family of proteins is the key in balancing pro- and anti-apoptotic stimuli. Anti-apoptotic proteins include Bcl-2, Bcl-xl and Bcl-w and pro-apoptotic examples are Bax, Bak and Bok [57].

DNA damage elicits intracellular and extracellular apoptotic responses mediated by p53, abl, c-myc, Rb and E2F. If anti-apoptotic factors do not stop these, there will be decreased mitochondrial membrane potential leading to cytochrome C release, oxidative stress, DNA fragmentation and the activation of caspases [58] and cell death. Cancer cells with high Bcl-2 expression may be less susceptible to apoptosis by cisplatin [59].

#### Protein damage

Apoptotic stimuli are not limited to DNA damage. Protein interactions with oxaliplatin have not been directly investigated, but platinum drugs have a high affinity to cellular proteins. Due to the resemblance of oxaliplatin and cisplatin, mechanisms of inducing apoptosis may be similar. The hydrophobic DACH moiety in oxaliplatin may facilitate drug interactions inside hydrophobic pockets of cellular proteins [59, 60].

Cisplatin adducts to DNA amount to approximately 10% and protein adducts 75-85%. Massive reactivity of platinum drugs with protein sulfhydryls is likely to distort the redox homeostasis of the cell sufficiently enough to trigger apoptosis. Thioredoxin has been implicated in cancer cell resistance to cisplatin. Cisplatin can inactivate thioredoxin and its regenerating enzyme thioredoxin reductase [60]. Faivre et al found that this enzyme can also be inhibited by oxaliplatin [31].

DNA and protein damage together may accelerate apoptosis [31]. The contribution of protein damage to apoptosis changed the belief that binding of a DNA reactive drug to proteins is merely a detoxification event [61, 62].

#### Role of p53

The tumor suppressor gene p53 is essential for normal growth, but it is present at almost undetectable levels in most cells [63, 64]. It regulates DNA replication, repair and recombination in order to eliminate DNA damage. It responds to DNA damage by up-regulating Bax synthesis and down-regulating Bcl-2 to control mitochondrial permeability and the progression of apoptosis. It translocates to the mitochondria and is sensitive to the levels of Bcl-2 and Bax there [65]. Mutation of p53 results in a malignant phenotype change which occurs in almost all cancers [64]. Its status is a modifier of platinum drug sensitivity. Dominant p53 mutations in ovarian cancer cells are a major contributor of cisplatin resistance [65]. Faivre et al demonstrated that p53 defective cells are not necessarily less sensitive to growth inhibition and apoptosis induction by oxaliplatin [31].

### Immunological mechanisms

The cause of death in cancer cells may be dependent on immunogenic or non-immunogenic signals and mechanisms. Immunogenic cell death initiated with changes on the cell surface and release of different mediators which results in cell death eventually. Dendritic cells (DCs) are antigen presenting cells which process antigen material and present it on its surface to T cell of the immune system. Defects in the immunogenic signals or in the immune effectors will result in treatment failure [9, 65].

Immunogenicities of cisplatin and oxaliplatin are different in spite of similarities between them in inducing immunogenic cell death (ICD). For example, oxaliplatin-treated cells interact with T cell and prime them for the production of interferon γ anti-cancer vaccination [9]. Cisplatin-treated cells cannot exhibit this mechanism.

Calreticulin (CRT) is multifunctional protein located in storage compartments associated with endoplasmic reticulum (ER). Different cancer cells cause production of CRT which are supposed to promotes macrophages to engulf and destroy these cancer cells; however, this whole process remains ineffective because these cancer cells also express CD47 which blocks CRT, therefore no macrophages are recruited to kill cancer cells. Antibodies to block CD47 may prove useful in cancer treatments in future. It has been recently demonstrated that anti-CD47 antibodies in mice model’s of myeloid leukemia and non-Hodgkin’s lymphoma were successful in eliminating the cancer cells without causing any damage to normal cells [66]. In the pre-apoptotic phase release of CRT and post apoptotic phases’ production of HMGB1 are required for ICD. Cisplatin and oxaliplatin are both found equally effective in producing these two proteins [67]. However, if either of them fails to induce signals for CRT or HMGB1 release, it will stop cell death [68]. Calreticulin induction may be one of the vital mechanisms immunogenically which may cause reduced efficacy of cisplatin in CRC patients [67].

Significant evidence is now available to indicate that colorectal cancer has gotten strong immunogenic bases. It has been demonstrated that when immunologically effecter cells, like CD3+ T cells, CD45RO+ T cells and macrophages, infiltrate colorectal cancer tissue, tumor progression is reduced [69].

Toll-like receptor 4 (TLR4) is a protein that in humans is encoded by the TLR4 gene [70]. It is involved in detection of bacteria and cancer cells and results in activation of the innate immune system. Colorectal cancers are immunogenic and oxapliatin has been found to cause on the cell surface, expression of immunogenic signals before the onset of apoptosis which activate innate immune system and results in T cell interferon γ production and interact with TLR4 of dendritic cells which create a tumor vaccine. Patients with mutant TLR4 genes have demonstrated decreased response to oxaliplatin in the treatment of metastatic cancer and their disease free survival span is also decreased [9]. Even a loss of functional TLR4 allele was found linked with decreased survival in colorectal cancer patients treated with oxaliplatin-based chemotherapy. Conversely this study demonstrated that TLR4 alleles should not affect the therapeutic response to cisplatin treatment; however, this finding needs more research to validate it [9, 65].

### Resistance

Resistance to platinum drugs develops in several ways. It develops either because of low availability of the drug intracellularly, increased detoxification of the drug inside the cell or because of the strong repair response from the cell to the damages incurred to it [71, 72].

It is not fully understood what the platinum drug uptake process inside the cell is. It is an energy-dependent process which is combined with an efflux pump as well. This complex mechanism of uptake and efflux does not let it become saturable [72]. This system of uptake and efflux has been evidently mentioned as the most common mechanism of resistance to cisplatin. Results of cisplatin resistance are extrapolated for oxapliatin as well [73].

The other most common resistance mechanism to cisplatin and oxaliplatin is increased glutathione concentration which effectively inactivates platinum compounds before DNA damage is induced. Metallothioneins are small cysteine rich proteins involved in metal detoxification and also potentially determine acquired platinum resistance. They may play a role as stress proteins in response to platinum complexes [74]. Once inside the cell platinum drugs are conjugated to glutathione. Enzymes involved in glutathione activity include glutathione S transferase (GST) and glutathione synthase (GS). Once conjugated, these platinum drugs are effluxed which increase drug resistance. GST is a marker of resistance to cisplatin. GST also plays a vital role in oxaliplatin resistance [75].

Other important mechanisms are related to DNA repair, by different enzyme repair systems like NER, MMR and post-replication repair. Enzymes involved in these systems if present in abundance, in other words “up-regulated” will make repair process more effective and increase drug resistance, for example, cells that overexpress ERCC1 are resistant to oxaliplatin [7]. Combining oxaliplatin with monoclonal antibodies may prevent or even reverse its resistance. *In vitro* assays demonstrated that cetuximab reduces the expression of NER components used to remove platinum DNA adducts [76].

Evidence is piling up that common gene variants (polymorphisms) may play substantial role in the DNA repair process and platinum conjugation. For example, gene coding is involved for the enzymes responsible in oxaliplatin accumulation, detoxicification and DNA adducts repair which may influence cellular response to oxaliplatin [77].

Deficiencies in apoptotic machinery are associated with cisplatin resistance. Cancer cells with high Bcl-2 expression are less susceptible to apoptosis by cisplatin [65]. However Gourdier et al found that the modulation of Bax, Bak and Bcl-xl expression is not involved in oxaliplatin resistance [78].

Therefore, it is quite obvious that resistance is a combination of different processes and each and every effort should be made to detail all of them and find ways around them to improve the cytotoxicity profile of these drugs.

## Toxicity

### Cisplatin

#### Nephrotoxicity

It has been demonstrated that cisplatin-induced nephrotoxicity is mainly caused by injury to renal epithelium, which may result in inflammatory responses and nuclear and mitochondrial DNA injury which activates cell death. In experimental animal models it has been demonstrated that platinum drugs-induced nephrotoxicity appears to be associated with oxygen free species which can be avoided by using free radical scavenging agents such as amifostine [79].

#### Neurotoxicity

Neurotoxicity in visual perception and hearing abilities start soon after the treatment commencement with cisplatin and can be assessed by using pre- and post-treatment nerve conduction studies [80]. It has been demonstrated that cisplatin non-competitively inhibits NHE-1, a membrane sodium hydrogen ion transporter [80], found on peripheral nerve cells of the ocular and aural stimuli receiving centers. This interaction is cisplatin-dose-dependent and reversible, and results in hydroelectric imbalances and cytoskeleton alterations [80].

#### Myelotoxicity

Cisplatin may be responsible for profound bone marrow suppression and hemolytic anemia [80].

### Oxaliplatin

#### The hematopoietic system

Oxaliplatin is found more myelotoxic than cisplatin and the severity of myelotoxicity is related to its dose. Neutropenia occurs in around 4% of the patients; however, hemolytic anemia and thrombocytopenia are usually not severe [81].

It has been suggested that oxaliplatin may affect bone marrow progenitor cells as its DNA adducts were found in leukocytes after treatment [82]. The real impact of this hematological toxicity is undefined; however, the amount of oxaliplatin DNA adducts in the blood cells of the patients may be related with their leucopoenia and thrombocytopenia severity [83].

It has also been noted that repeated oxaliplatin infusions may result in hypersensitivity reactions which could consequently result in hemolytic anemia and secondary immune thrombocytopenia [84]. Occasionally rare cases of secondary acute leukemia have also been reported [85].

#### Neurotoxicity

Peripheral neuropathy is the common side effect of oxaliplatin treatment and it could be acute or chronic. Acute peripheral neuropathy can manifest itself as paresthesia, dysthesia, or allodynia of the extremities, lips and orolarynogopharynx during or immediately after the treatment [86]. Studies have demonstrated that oxalate a metabolite of oxaliplatin interacts with voltage-gated sodium channels in complex pathways involving calcium, as a result, calcium gets chelated [87] which may block the conduction pathways and result in peripheral neuropathy. It mainly involves sensory fibers rather than motor fibers.

Repeated oxaliplatin infusions may culminate in chronic peripheral neuropathy which exhibits as decreased distal sensations and proprioception. Fifteen percent of the patients receiving oxaliplatin’s cumulative dose of approximately 800 mg/m^2^ can suffer with grades 3 and 4 neuropathy [88]. Initially the theory regarding the pathophysiology was that it happens because of a degenerative process of the axons; however, currently it has been postulated that accumulation of oxaliplatin in the dorsal root ganglia cells results in their atrophy and mitochondrial dysfunction which results in neurotoxicity [89]. Fortunately it is found reversible in the majority of the cases except in around 5% of patients and like, its acute counterpart it mainly involves sensory fibers rather than motor fibers which are rare [9].

## Discussion

As insights in molecular biology of the cancers are increasing, it is opening up new vista of treating them and brining new pharmacological combinations in place to provide an effective and less toxic treatment.

The standard chemotherapy drugs work by stopping the cell division with limited selectivity, therefore in doing that they also disrupt the division of normal cells in combination of cancers cells [90]. This unselectivity severely damages the rapidly growing non-cancerous cells, which limits the efficacy of the anticancer drug or drug regimens [91] which plays a part in patients’ poor quality of life and drug intolerability. It may have a role in increasing drug resistance as well [67].

MADs address this problem of unselectivity and act on cancer cells specifically. For example, MAD, cetuximab selectively binds with extracellular domain of epidermal growth factor receptor (EGFR) [92, 93], similarly trastuzumab binds with the extracellular domain of human epidermal growth factor receptor 2 (HER2) [94, 95] and bevacizumab binds with VEGF [96]. All these bondages selectively happen with cancer cells and block the specific actions of these receptors/proteins.

Cisplatin has gotten a proven record in treating testicular cancers and oxaliplatin in colorectal cancers; however, neither of them selectively target cancer cells and their toxicity profile is not promising either. Therefore the need to combine them with MAD to increase their cancer specific cytotoxicity and decrease in their toxicity profile is the way forward for future chemotherapeutic regimens. For example, combining bevacizumab with folfox or xelox for metastatic CRC demonstrated good response rates and increased disease progression-free overall survival [97]. However, the beneficial effects of these MADs are restricted to colorectal cancer patients which were diagnosed with unmutated KRAS gene in their cancers [9, 98].

Therefore, it is imperative that MADs which cannot be used as monotherapy, their combination with conventional agents should be based on rational and scientific combinations. This rational combination would come from the understanding of the mechanism of actions of conventional and MAD separately and in combinations through studies which are designed to address pharmacological and clinical development of these drugs. It is thus valuable to revisit the molecular mechanisms of conventional chemotherapeutic agents which will assist in designing new complementary and synergistic combination regimens for future trials [7].

Molecular predictive markers are also under investigation and require prospective, hypothesis-driven and randomized clinical trials. Only a few molecular predictors have already entered clinical use. This may change in the near future and the majority of therapeutic decisions will account for genetics [99].

## Conclusion

Understanding the mechanisms of action and resistance of cisplatin and oxaliplatin will facilitate designing of future clinical trials with MAD combinations which will improve their cytotoxicity profile, reduce their toxicities and improve treatment outcome, which will result in better tolerability and patient satisfaction.
